# A Nonintegrative Lentiviral Vector-Based Vaccine Provides Long-Term Sterile Protection against Malaria

**DOI:** 10.1371/journal.pone.0048644

**Published:** 2012-11-02

**Authors:** Frédéric Coutant, Raul Yusef Sanchez David, Tristan Félix, Aude Boulay, Laxmee Caleechurn, Philippe Souque, Catherine Thouvenot, Catherine Bourgouin, Anne-Sophie Beignon, Pierre Charneau

**Affiliations:** 1 Unité Virologie Moléculaire et Vaccinologie, Department of Virology, Institut Pasteur and CNRS URA3015, Institut Pasteur, Paris, France; 2 Centre de Production et d’Infection des Anophèles (CEPIA), Department of Parasitology and Mycology, Institut Pasteur, Paris, France; Naval Medical Research Center, United States of America

## Abstract

Trials testing the RTS,S candidate malaria vaccine and radiation-attenuated sporozoites (RAS) have shown that protective immunity against malaria can be induced and that an effective vaccine is not out of reach. However, longer-term protection and higher protection rates are required to eradicate malaria from the endemic regions. It implies that there is still a need to explore new vaccine strategies. Lentiviral vectors are very potent at inducing strong immunological memory. However their integrative status challenges their safety profile. Eliminating the integration step obviates the risk of insertional oncogenesis. Providing they confer sterile immunity, nonintegrative lentiviral vectors (NILV) hold promise as mass pediatric vaccine by meeting high safety standards. In this study, we have assessed the protective efficacy of NILV against malaria in a robust pre-clinical model. Mice were immunized with NILV encoding *Plasmodium yoelii* Circumsporozoite Protein (Py CSP) and challenged with sporozoites one month later. In two independent protective efficacy studies, 50% (37.5–62.5) of the animals were fully protected (p = 0.0072 and p = 0.0008 respectively when compared to naive mice). The remaining mice with detectable parasitized red blood cells exhibited a prolonged patency and reduced parasitemia. Moreover, protection was long-lasting with 42.8% sterile protection six months after the last immunization (p = 0.0042). Post-challenge CD8+ T cells to CSP, in contrast to anti-CSP antibodies, were associated with protection (r = −0.6615 and p = 0.0004 between the frequency of IFN-g secreting specific T cells in spleen and parasitemia). However, while NILV and RAS immunizations elicited comparable immunity to CSP, only RAS conferred 100% of sterile protection. Given that a better protection can be anticipated from a multi-antigen vaccine and an optimized vector design, NILV appear as a promising malaria vaccine.

## Introduction

*Plasmodium* is the causative agent of malaria, a life-threatening disease affecting 216 million people worldwide and responsible for 655 000 deaths in 2010 according to the *World malaria report 2011.* Repeated childhood exposure to *Plasmodium* naturally confers specific immunity that protects against the most severe forms of malaria, but does not confer sterile protection. Children remain at risk until they have developed this partial immunity [1]. Therefore an ideal malaria vaccine should fully prevent infection from early infancy onwards.

*Plasmodium* sporozoites are inoculated into the host’s skin by bites from infected mosquitoes. After invading skin blood vessels, they migrate to the liver where they invade hepatocytes and develop. Infected hepatocytes then produce and release merozoites into the blood circulation, which in turn invade red blood cells [2], [3], [4]. The liver-stage is asymptomatic while the erythrocytic stage is pathogenic. Immunizations with radiation-attenuated sporozoites (RAS), which interrupt their development inside hepatocytes, can confer sterile protection against malaria in humans [5] and rodents [6]. However, this strategy is not easily applicable to large-scale approaches because of major technical and logistical limitations, and was sub-optimally immunogenic and protective in a recent phase I/IIa trial following subcutaneous and intradermal injections [7]. Several other candidate vaccines, such as adenovirus or poxvirus vectorized Ag, have been or are being evaluated for safety and immunogenicity and then for protection using experimental challenges or in-field trials [8], [9], [10]. Malaria vaccine projects at advanced pre-clinical and clinical stages globally are summarized by the WHO (the WHO.28_Nov_2011 Malaria Vaccine Rainbow Tables http://www.who.int/vaccine_research/links/Rainbow/en/index.html). However, vaccine-induced immunity has so far failed to confer strong and long-lasting protection against malaria [11], [12]. The most advanced candidate vaccine is the RTS,S, a sub-unit vaccine based on a single pre-erythrocytic antigen (Ag), the Circumsporozoite protein (CSP) from *Plasmodium falciparum (Pf)*. It was shown to substantially reduce clinical and severe *Pf* malaria episodes in infants from seven countries in sub-Saharan Africa in a large phase III clinical trial yet without completely preventing infection [13]. Longer-term protection needs to be documented and higher rates of protection are likely required to achieve eradication of malaria in endemic zones [14].

Thus, there is an urgent need to develop new vaccine strategies, including new vectors. The liver stage, although clinically silent, plays a key role in the parasite life cycle. A vaccine aiming to block *Plasmodium* at the early steps of its cycle in the vertebrate host is likely to be more successful than a vaccine based on erythrocytic Ags only. A mosquito bite delivers about 100 sporozoites in the skin. It results in the rapid invasion of few hepatocytes [15], [16]. The liver-stage is completed in a few days, depending on the parasite and host. Then, each infected hepatocyte releases about 30,000 merozoites into the blood stream [17]. Infection of hepatocytes renders parasites susceptible to recognition and elimination by CD8^+^ T cells [18]. However, the low number of sporozoites, the low frequency of infected hepatocytes and the short duration of the liver stage make the task considerably difficult for neutralizing Abs and/or effector T cells. It is anticipated that a high frequency of CD8+ T cells with immediate effector functions in the liver is required for protection against the disease [19], [20], [21], [22].

HIV-1 derived lentiviral vector (LV) are very potent at inducing strong and broad cellular and humoral memory responses [23], [24], [25], [26], [27]. They provide protective immunity against many tumors and infectious diseases as shown in mice and monkeys [28], [29], [30], [31]. These properties are explained by their adjuvanticity [32], [33], [34] and ability to efficiently transduce non-dividing cells and in particular dendritic cells (DC), which are the most effective antigen-presenting cells [35], [36], [37]. Despite these advantages, their integrative status challenges their safety profile. The risk of insertional mutagenesis likely precludes their large-scale use as prophylactic and pediatric vaccines. Insertional mutagenesis results from the presence of transcriptional enhancer sequences within the vector construct [38], [39], [40], therefore the use of promoters devoid of associated enhancer activity is a means to improve the safety of integrative LV (ILV) [41]. However, one of the best strategies to obviate the risk of insertional oncogenesis is to eliminate the integration step altogether by using a nonintegrative LV (NILV) carrying a defective HIV-1 integrase [42]. Double-stranded episomal DNA circles, which accumulate in the nucleus as a result of the integration defect, are highly competent for transcription. Hence, transduction with NILV leads to the potent and sustained expression of the gene of interest and effective gene therapy for post-mitotic tissues such as ocular and brain tissues or liver [43], [44], [45], [46]. In contrast to ILV, NILV mediate stable gene expression only in non-dividing cells, whereas expression is transient in proliferating cells because of the partition of the episomes between daughter cells and progressive dilution as cells further divide. Since DC are non-dividing highly differentiated cells, NILV should be immunogenic. We have shown that NILV transduce conventional and plasmacytoid murine DC as efficiently as ILV and that immunization with NILV encoding a secreted form of the envelope of West Nile Virus protects mice against lethal challenge through the induction of neutralizing antibodies [47]. It was also reported that mice immunized with NILV mount potent CD8+ T cells against various antigens, such as HIV-1 gp120 and gag, HBsAg or OVA and hgp100, which mediate effective tumor prophylaxis and therapy [48], [49], [50], [51], [52], [53].

To explore the protective efficacy conferred by NILV against malaria, we used the major pre-erythrocytic stage malaria vaccine candidate Ag, CSP. CSP is the main protein of invading sporozoites and it is highly immunogenic. It continues to be transcribed in liver cells, and is very potently presented by infected cells on their MHC class I molecules [18], [54]. RTS,S, which is composed of a single Ag, *Pf* CSP, provides some protection against clinical and severe malaria [13].

We performed challenge experiments of BALB/c mice with *Plasmodium yoelii* (*Py*). In this murine model of malaria, which is widely used for the pre-clinical development of vaccines and drugs, both antibodies to the central repeat domain of CSP and CSP-specific CD8^+^ T cells have been shown to mediate protection, by inhibiting the migration of sporozoites from the skin to the liver as well as hepatocyte invasion and by hindering the development of parasites within hepatocytes, respectively, thus preventing blood-stage malaria [55], [56], [57], [58].

Here we report that a NILV encoding CSP induce protective CD8+ T cell responses against malaria. After three immunizations, 50% (37.5–62.5) of the animals were fully protected when the challenge with sporozoites was carried out one month after the last immunization as demonstrated in two independent challenge studies, while 42.8% of the mice did not develop parasitemia when challenged 6 months after the last immunization. In addition to this sterilizing and long-term protection, the remaining vaccinated animals with detectable parasitized red blood cells exhibited a delayed erythrocyte infection compared with naive animals and a reduced parasitemia. Since immune control is exerted at the pre-erythrocytic stage with CSP as the sole targeted Ag, this suggested a reduced parasite burden in the liver.

To our knowledge, this is the first report of the use of LV as malaria vaccine candidate. Data are encouraging. They provide a proof-of-concept for the protective efficacy against malaria with a basic NILV. Studies are ongoing to discover new protective Ags and to improve the design of NILV to ensure stronger immunogenicity and higher rates of protection.

## Results

### Comparison of the Immunogenicity of Integrative and Nonintegrative Lentiviral Vectors

**Figure 1:**
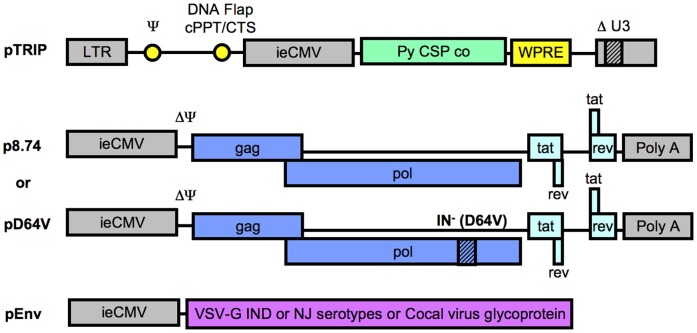
Nonintegrative lentiviral vector encoding *Plasmodium yoelii* CSP used in the study. Lentiviral vector particles were produced by transient transfection of 293 T cells. The three plasmids used to generate particles are represented here (schematic representation not to scale). The vector expression plasmid pTRIP encodes the vaccine antigen, *Plasmodium yoelii* CSP. The encapsidation plasmid, p8.74 or pD64V for ILV or NILV respectively, codes for HIV-1 proteins required for particle formation and transduction. The envelope expression plasmid encodes non-crossreacting glycoproteins from Vesiculoviruses used in a specific order to circumvent anti-vector particles antibodies generated after each immunization (Vesicular Stomatitis Virus glycoprotein (VSV-G) Indiana (IND) serotype followed by VSV-G New Jersey (NJ) serotype followed by Cocal virus glycoprotein). Genes coding for structural/enzymatic and regulatory HIV-1 proteins are in dark and light blue respectively, while HIV-1 *cis*-acting sequences are in yellow and promoter sequences are in grey. The transferred gene, *Py* CSP, with a human codon-optimized sequence is in green. LTR, long terminal repeat; Ψ, encapsidation signal; cPPT/CTS, central polypurine tract/central termination sequence responsible for the formation of the DNA Flap structure during reverse-transcription which is a determinant of HIV-1 nuclear import; WPRE, Woodchuck hepatitis virus post-transcriptional response element to enhance mRNA nuclear export on a Rev/RRE independent fashion.

**Figure 2:**
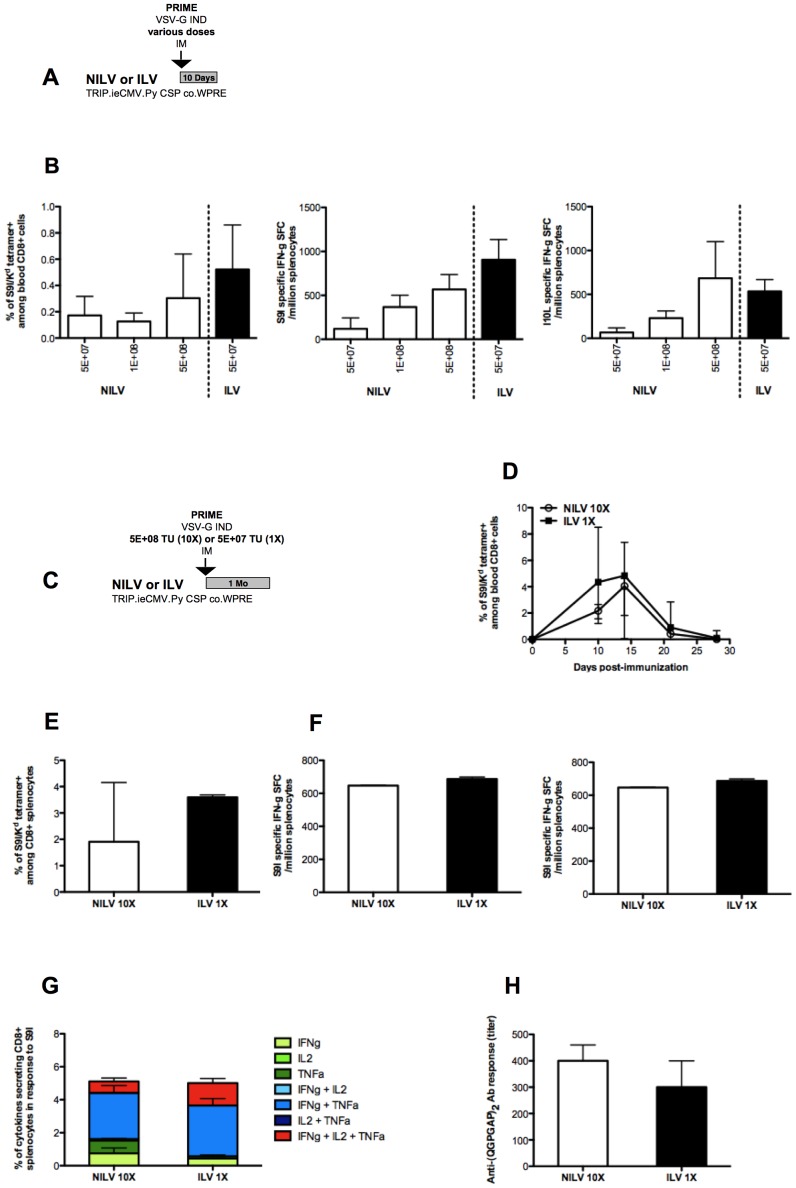
NILV are as immunogenic as ILV when 10-times more particles are injected. BALB/c mice (n = 5/group) were immunized by IM injection with various doses (expressed as TU/mouse) of lentiviral vector particles encoding Py CSP, either NILV (□) or ILV (▪). Ten days later, specific cellular immune responses were assessed (**Figure 2A**). The frequency of S9I-specific blood CD8+ cells was assessed by S9I/K^d^ tetramer staining, and the frequency of IFNg secreting splenocytes in response to overnight restimulation with S9I or I10L peptides was measured by IFNg elispot assay (**Figure 2B**). Means + SD are shown. BALB/c mice (n = 3/group) were IM immunized with NILV (□) or ILV (▪) at the dose of 5E+08 or 5E+07 TU/mouse respectively (**Figure 2C**). The frequency of S9I-specific blood CD8+ cells was followed over time by tetramer staining (**Figure 2D**). At day 24 post-immunization, spleen cellular response was analyzed by S9I/K^d^ tetramer staining (**Figure 2E**), by IFNg elispot in response to S9I and I10L peptides (**Figure 2F**), and by intracellular staining of 3 cytokines, IFNg, IL2 and TNFa, in response to S9I (**Figure 2G**). Cells secreting individual (green), 2 (blue) or 3 (red) cytokines are shown. Anti-(QGPGAP)_2_-specific IgG at day 21 post-immunization were quantified by ELISA and expressed as titers (**Figure 2H**). Medians + range are shown.

We first compared the intensity of cellular immune responses induced by NILV and ILV. Both types of vector particles are produced by transient transfection of 293 T cells. They only differ by the D64V substitution in the catalytic domain of the HIV-1 integrase encoded by Pol, blocking the DNA cleaving and joining reactions of the integration step as previously described [59] (**Figure 1**). The cellular immune responses directed against two CD8+ T cells immunodominant epitopes present in *Py* CSP, S9I and I10L [60], [61], were assessed 10 days after a single injection of various doses of vector particles (**Figure 2**). Immunization with NILV resulted in lower frequencies of S9I-specific blood CD8+ T cells (quantified by tetramer staining) and of S9I- and I10L-specific IFNg secreting splenocytes (measured by IFNg elispot) as compared to ILV at the same dose, 5E+07TU/mouse (**Figure 2B**). The lower immunogenicity of NILV compared to ILV was not dependent on CSP, as this was also observed with an unrelated Ag, SIV GAG (**Figure S2**); and it could be compensated by increasing the dose of vector particles (5E+08TU/mouse) (**Figure 2B**).

We next further characterized immune responses induced by 5E+08 TU/mouse of NILV *versus* 5E+07 TU/mouse of ILV (**Figure 2C**). The kinetics of blood responses were quite similar (**Figure 2D**). About one month after immunization, spleen cellular immune responses induced by 10-times more NILV particles could not be distinguished quantitatively and qualitatively from those induced by ILV, as tested by tetramer staining (**Figure 2E**), IFNg elispot (**Figure 2F**) and intracellular staining of cytokines (ICS for IFNg, IL2 and TNFa) (**Figure 2G**). The humoral response against CSP, measured 3 weeks post-immunization, was also found to be comparable between ILV and a 10-times higher dose of NILV (**Figure 2H**).

In conclusion, an increased dose of vector particles could overcome the relative defect of immunogenicity of NILV compared to ILV, and a potent cellular and humoral immunity against CSP could be elicited. The use of NILV was thus validated. NILV offer the important advantage of circumventing any fear about safety issues related to insertional mutagenesis.

### Comparison of Immune Responses Directed against CSP after Immunizations with Nonintegrative Lentiviral Vector Particles and Radiation-attenuated Sporozoites

**Figure 3:**
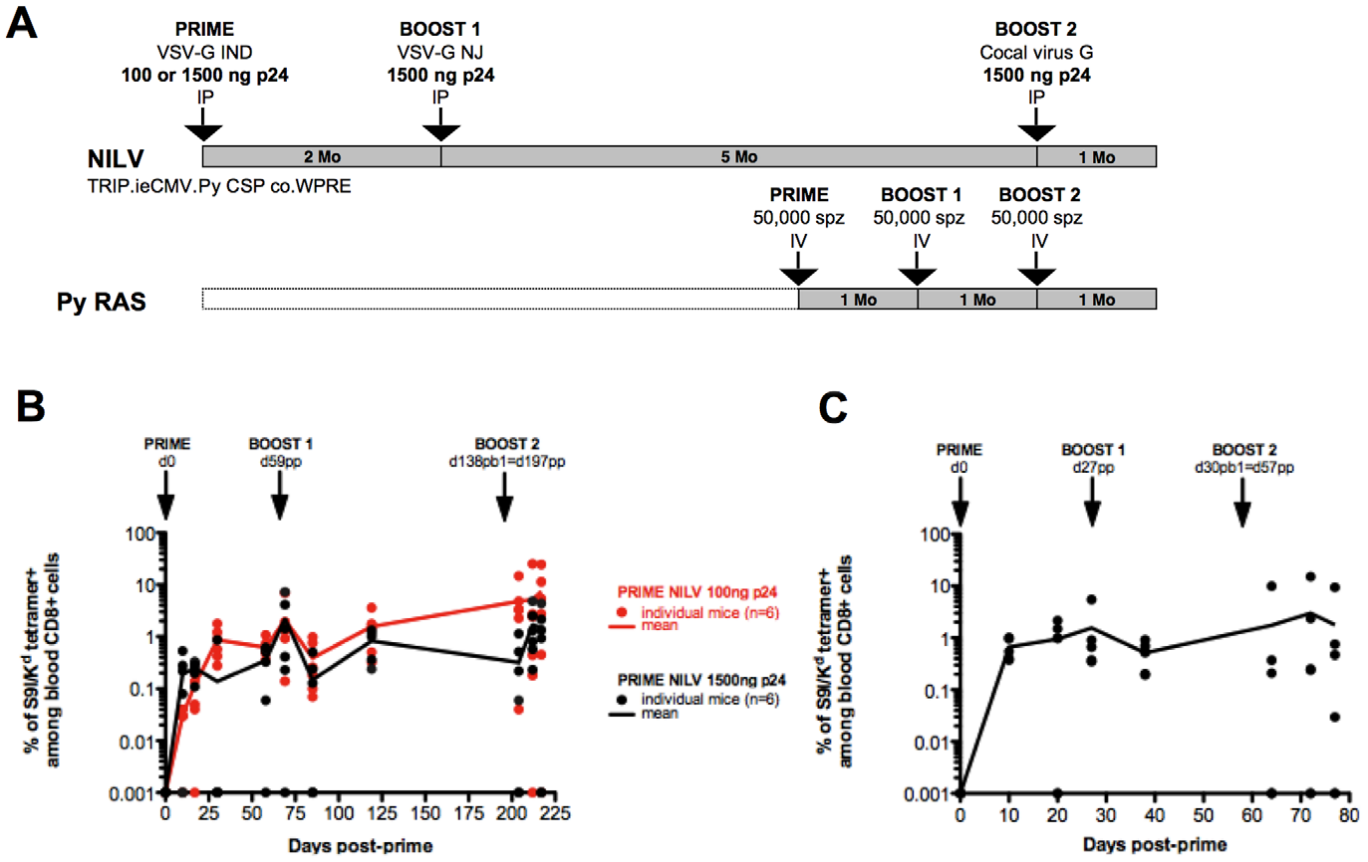
NILV elicit as frequent blood CSP-specific T cells as RAS after 3 injections. BALB/c mice (n = 6/group) were immunized 3 times by IP injections of NILV. They were primed by administration of NILV particles encoding Py CSP and pseudotyped with VSV-G IND at the dose of 100 or 1500 ng p24/mouse. They were boosted 2 months later with 1500 ng p24 of NILV particles pseudotyped with VSV-G NJ, and boosted again 5 months later with 1500 ng p24 of NILV particles pseudotyped with the glycoprotein from Cocal virus. Additionally, mice (n = 6) from the same batch were immunized 3 times by IV injection with RAS at monthly intervals (**Figure 3A**). The frequency of S9I-specific blood CD8+ cells was followed over time by S9I/K^d^ tetramer staining after NILV (**Figure 3B**) and RAS immunizations (**Figure 3C**). Data from individual mice and means are shown. The Y-axis uses a logarithmic scale.

Multiple injections of RAS are known to induce potent sterilizing immunity in mice and are rightly considered as the gold standard of protection against *Plasmodium* infection. A prime/boost strategy was designed to compare vaccine efficacy induced by NILV and RAS. Vector particles were pseudotyped with non-cross-reactive envelopes to allow efficient *in vivo* iterative administrations (**Figure 1**). Mice received three successive injections of NILV particles pseudotyped with the glycoprotein of Vesicular Stomatitis Virus (VSV-G) serotype Indiana (IND) first, then the VSV-G serotype New Jersey (NJ) and finally the glycoprotein from the Cocal Vesiculovirus. For the first injection, two doses were tested, either 100 ng p24 or 1500 ng p24 (corresponding to 1.48E+07 or 2.22E+08 TU/mouse respectively for this batch of vector). The first boost was performed 2 months after the prime immunization with 1500 ng p24 (2.88E+08 TU/mouse), while the second boost was done 5 months after the first one, also with 1500 ng p24 (3.33E+08 TU/mouse). For RAS immunization, mice were immunized three times with 50,000 irradiated sporozoites at monthly intervals (**Figure 3A**). The frequency of blood specific T cells was followed longitudinally by tetramer staining. For NILV, a priming dose lower than the boosting doses (100–1500–1500 ng p24) led to more specific T cells than three injections with 1500 ng p24 (**Figure 3B**). This prime/boost protocol also induced as many blood CSP specific T cells as three injections of RAS (**Figure 3C**). Thus it was selected for our detailed comparative functional analysis.

**Figure 4:**
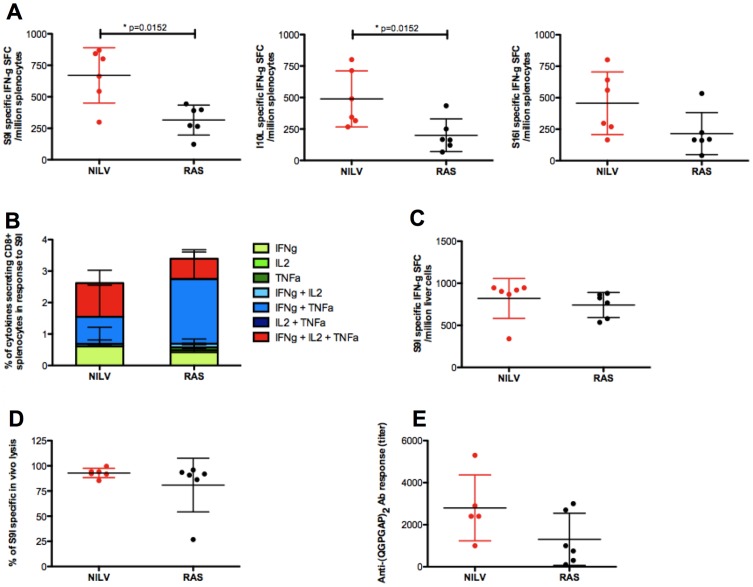
Three immunizations with NILV and RAS induce comparable pre-challenge CSP-specific immune responses. Groups of BALB/c mice (n = 6/group) were immunized 3 times with NILV by IP injections (in red) or with RAS by IV injections (in black). Immune responses, both cellular and humoral, were compared 28 days after the last immunization. The frequency of IFNg secreting splenocytes in response to restimulation with S9I, I10L or S16I peptides was measured by IFNg elispot assay (**Figure 4A**). The quality of the S9I specific response was further studied by intracellular staining of 3 cytokines, IFNg, IL2 and TNFa, in response to S9I (**Figure 4B**). The frequency of IFNg secreting liver cells after restimulation with S9I was analyzed by IFNg elispot (**Figure 4C**). Additional mice (n = 6/group) were immunized to compare the vaccine-induced *in vivo* killing capacity of S9I-pulsed target cells (**Figure 4D**). The presence of IgG directed against (QGPGAP)_2_ was assessed by ELISA (**Figure 4E**). Individual responses, means and SD are shown.

One month after the last immunization, responses towards S9I, I10L and S16I (a peptide containing a Py CSP CD4+ epitope and the S9I CD8+ T cell epitope) were measured in the spleen by IFNg elispot (**Figure 4A**) and by ICS (**Figure 4B**). They were of the same order of magnitude or even a bit higher with NILV than with RAS immunizations. T cells were multifunctional and able to produce simultaneously IL2, IFN-γ and TNF-α. When liver cells were re-stimulated with S9I, their IFNg responses were similarly high (**Figure 4C**). When target cells pulsed with S9I were injected in mice immunized with NILV or RAS, they were promptly and equally well killed *in vivo* (**Figure 4D**). Finally, both NILV and RAS immunizations led to an equivalent generation of antibodies to CSP (**Figure 4E**). Collectively, this comparison study demonstrated that three injections of NILV result in immune responses directed against CSP, which were comparable in intensity and quality with three injections of RAS.

### Comparison of the Protective Efficacy of Nonintegrative Lentiviral Vectors and Radiation-attenuated Sporozoites against Malaria

One month after the last boost, animals immunized with NILV (100–1500–1500ng p24) and RAS were challenged intravenously with 500 live sporozoites. Protection was evaluated by monitoring the duration of the pre-patent period (delay between challenge and appearance of blood stage parasites) and parasitemia (the percentage of parasitized red blood cells). Full protection was defined as the complete absence of parasites in blood after sporozoite challenge (sterile immunity). Immune control is exerted at the pre-erythrocytic-stage of the life cycle of the parasites since CSP is targeted. It implies that a delay in the pre-patent period and onset of blood-stage infection results from a reduced parasite burden in the liver. This was considered as partial vaccine efficacy.

**Figure 5:**
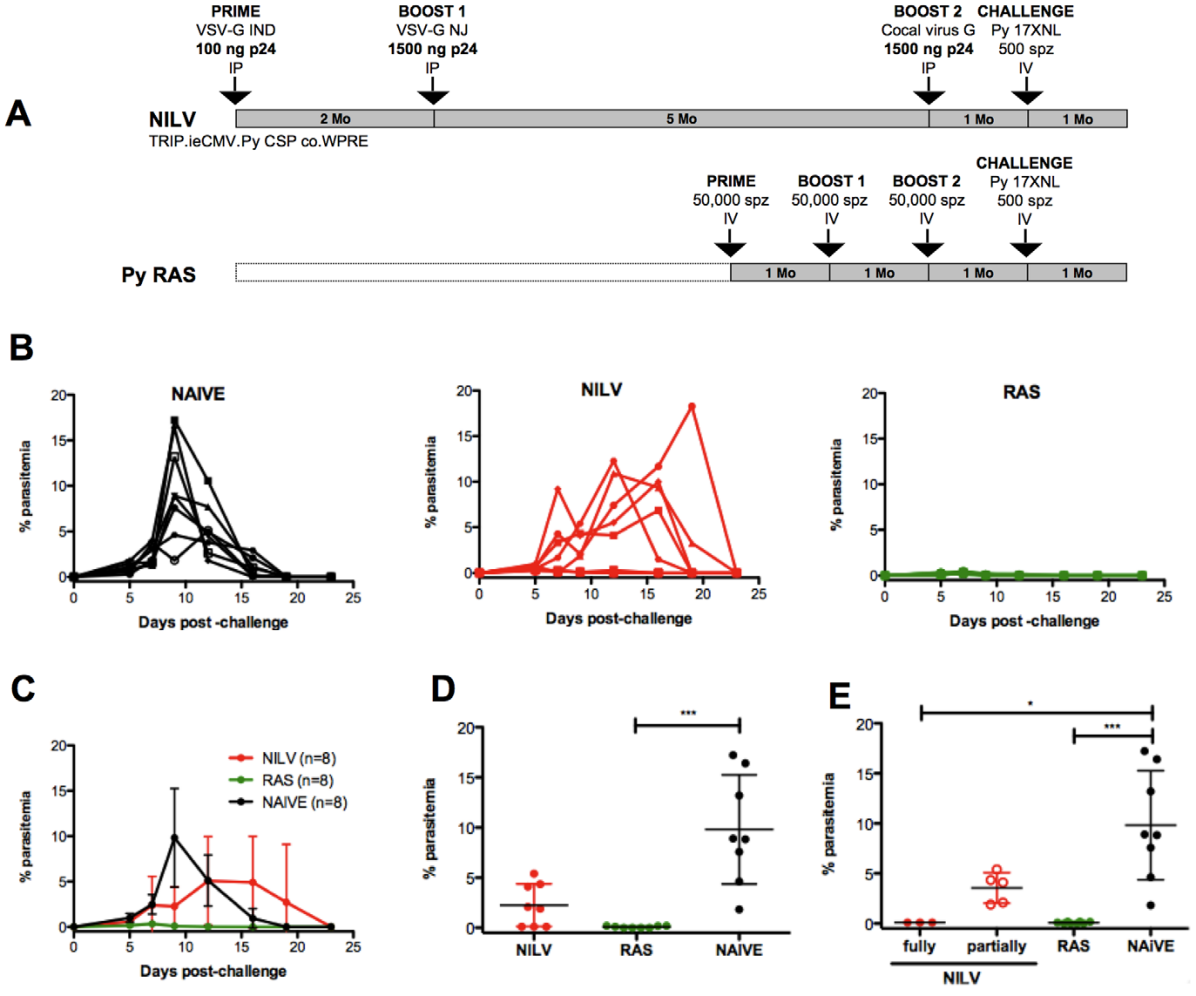
NILV immunizations provide protection against parasitemia after sporozoites challenge, but not as strong as RAS. Groups of BALB/c mice (n = 8/group) were immunized 3 times with NILV by intraperitoneal injections (in red) or with RAS by intravenous injections (in green), or not (in black) (**Figure 5A**). They were challenged with 500 spz injected IV 28 days after the last immunization. The protective efficacies of both vaccines against malaria were compared. Parasitemia were followed every other day from day 5 to day 23 post-challenge by Giemsa-stained blood smears. The longitudinal follow-up of individual parasitemia is shown (**Figure 5B**) as well as means + SD (**Figure 5C**) and parasitemia at day 9 post-challenge (which corresponds to the peak of viremia in the group of naive animals) (**Figure 5D**). Among the NILV-immunized mice (in red), fully (•) *versus* partially (○) protected animals were further distinguished (**Figure 5E**). The Kruskal-Wallis test was used to compare 3 or 4 groups (Figure 5D and Figure 5E respectively), followed by a Dunn’s multiple comparison post-test. Asterisks denote significance for the post-test (*p<0.05, **p<0.01 and or ***p<0.001). When comparing NILV and naive mice with a Mann-Whitney test (**Figure 5D**), **p = 0.0072.

After 5 days, all naive mice exhibited patent parasitemia. By contrast, some vaccinated animals were fully protected (**Figure 5A**). Sterile immunity was observed in 37.5% of the mice immunized with NILV (3 out of 8 animals) and in 100% of the mice immunized with RAS. Moreover, in the 5 remaining NILV immunized mice with detectable parasitemia, there was a delay in the course of erythrocyte invasion, as well as a 2.75 fold reduction in the level of parasitemia compared to naive animals at day 9 post-challenge (**Figures 5B–5E**).

**Figure 6:**
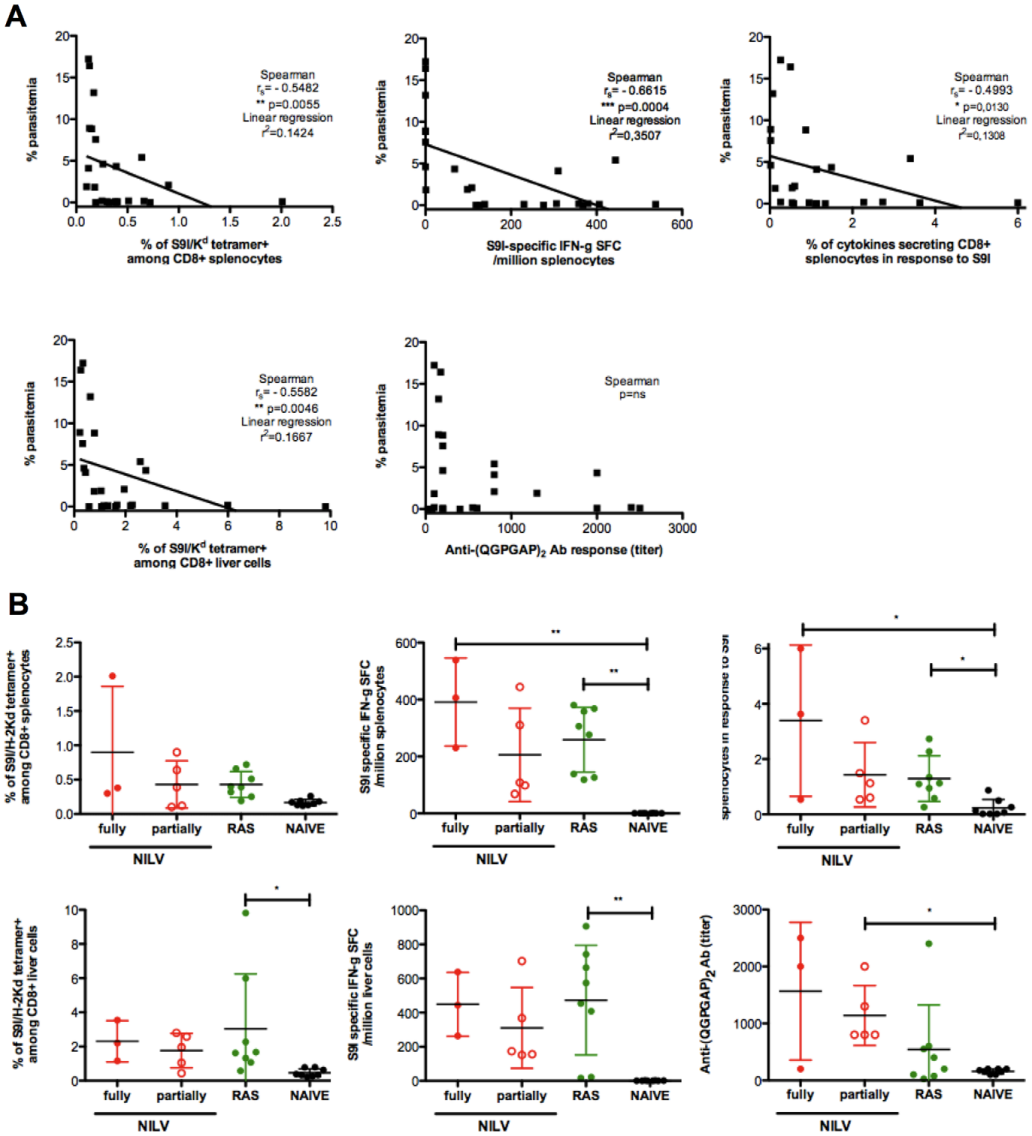
Protection is associated with CSP-specific CD8+ T cells responses. Immune correlates of protection against malaria were studied by plotting day 28 post-challenge immunity and day 9 post-challenge parasitemia as X and Y variables and using the Spearman test (the r_s_ and p values are shown) and linear regression (r^2^ is shown) (**Figure 6A**). Immune responses in challenged mice were compared 28 days post-challenged between the vaccine candidates and their level of protection (fully (•) or partially (○) protected NILV immunized animals in red) by S9I/K^d^ tetramer staining and IFNg elispot assay with splenocytes and liver cells and elisa (**Figure 6B**). Means and SD are shown. The Kruskal-Wallis test was used to compare 3 or 4 groups, followed by a Dunn’s post-test.

We next sought to elucidate immune correlates of protection. We compared day 28 post challenge immune responses (day of euthanasia) with day 9 parasitemia (peak of parasitemia for the naive animals). Analysis of immune responses in challenged animals revealed that CSP-specific CD8+ T cells correlated with the levels of protection against infection but not with anti-CSP Abs (**Figure 6A**). Among the various immune functions directed against CSP that we analyzed, none allowed to distinguish RAS- and NILV-vaccinated protected mice (**Figure 6B**).

**Figure 7:**
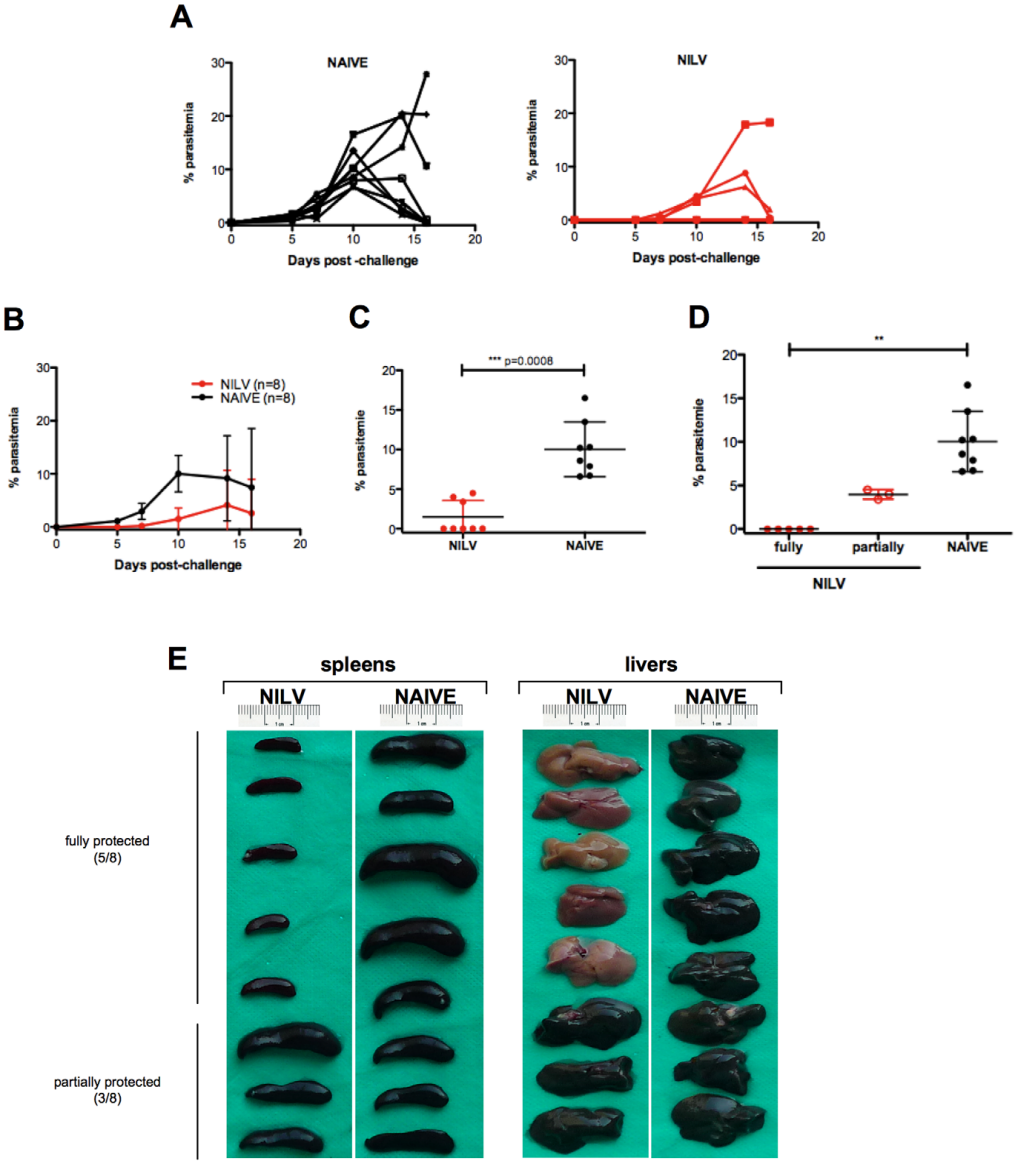
The protective efficacy of NILV was confirmed in a second independent study. Groups of BALB/c mice (n = 8/group) were immunized 3 times with NILV by intraperitoneal injections (in red) or not (in black). They were challenged with 500 spz injected IV one month after the last immunization. The % of parasitized red blood cells was followed every other day from day 5 to day 16 post-challenge by Giemsa-stained blood smears. Individual parasitemia are shown (**Figure 7A**) as well as means + SD (**Figure 7B**) and parasitemia at day 10 post-challenge (**Figure 7C**). Among the NILV-immunized mice (red circles), fully (•) *versus* partially (○) protected animals were further distinguished (**Figure 7D**). The Mann-Whitney test was used to compare NILV and naive and the Kruskal-Wallis test followed by a Dunn’s post-test were used to compare fully, partially and naive. The gross morphology of spleens and livers from NILV-immunized and naive mice at necropsy were compared 3 weeks post-challenge (**Figure 7E**).

These data showing that NILV immunizations can provide a potent immune control of the parasite liver stage were strengthened by a second independent study. In this trial, NILV afforded an even stronger protection, with 62.5% sterile protection (5 out of 8 mice) (**Figures 7A–7B**). Partially protected mice also showed two times less parasitized red blood cells in comparison with naive animals (**Figures 7C–7D**). As expected three weeks post-challenge, naive mice displayed a dramatic splenomegaly. Moreover, their spleens and livers showed a dark pigmentation likely resulting from the accumulation of hemozoin produced by the parasite during the digestion of red blood cell hemoglobin. By contrast, the capacity of 5 out of 8 vaccinated mice to mount a sterile immune response coincided with the preservation of their spleen size and liver pigmentation (**Figure 7E**).

**Figure 8:**
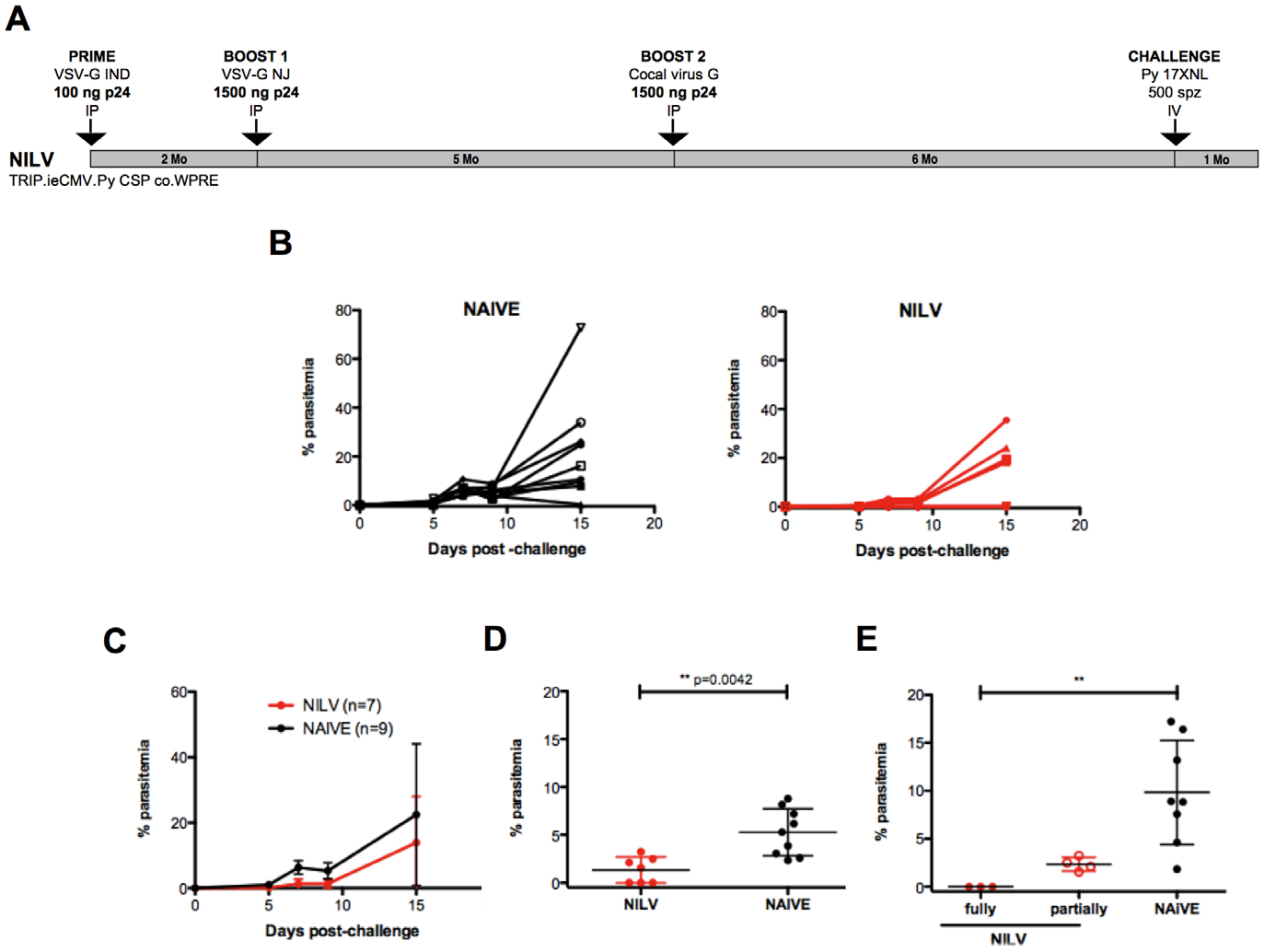
NILV immunizations elicit enduring protective memory responses against malaria. Groups of BALB/c mice were immunized 3 times with NILV by intraperitoneal injections (n = 7) (in red) or not (n = 9) (in black). They were challenged with 500 spz injected IV six months after the last immunization (**Figure 8A**). The % of parasitized red blood cells was followed by Giemsa-stained blood smears. Individual parasitemia are shown (**Figure 8B**) as well as means + SD (**Figure 8C**) and parasitemia at day 9 post-challenge (**Figure 8D**). Among the NILV-immunized mice (in red), fully (•) *versus* partially (○) protected animals were further distinguished (**Figure 8E**).

Importantly, even when the challenge was performed 6 months after the last immunization with NILV (**Figure 8A**), 42.8% of the mice still failed to develop any detectable parasitemia, while the remaining vaccinated mice succeeded in controlling parasitemia to a lower level compared to naive animals (**Figures 8B–8E**). This important result illustrates the long-lasting protection conferred by our vaccine strategy.

## Discussion

Our studies were designed to define the protective efficacy of a LV based-candidate malaria vaccine. Because NILV resolve the much-feared risk of integrase-mediated insertional mutagenesis, they would preferentially be used over ILV as prophylactic and pediatric vaccine. Integration events in cells transduced with NILV are limited to illegitimate recombination [42]. They are expected to be as extremely rare as with other transient gene delivery methods such as DNA vaccines, which have a good clinical safety record.

We report here that a single injection of NILV encoding CSP elicits potent and sustained specific T cells. However, it appears that current NILV are not as good as their integrative counterpart to induce T cell responses and 10-times more NILV particles were required to obtain immune responses as strong as with ILV, as described previously [49], [51]. This might be due either to the intensity of Ag expression, which would be insufficient with NILV compared to ILV, and/or to the critical involvement of some dividing cells in the induction of immunity by LV since mitotic cells lose episomal DNA contrary to integrated DNA.

A prime injection with a low dose of NILV followed by two boosts with a higher dose led to CSP-specific immune responses similar to three injections of RAS. Of note, responses to CSP induced by RAS are usually moderate compared to other CSP-based strategies, such as adenovirus or poxvirus [8], [62], [63]. One month after the last immunization, NILV induced IFN-g secreting cells in the spleen and liver (744±137 and 800±259 S9I specific IFN-g SFC/million respectively). Cells were poly-functional (simultaneous secretion of IL2, IFNg and TNFa in response to SI9 by 1.12±0.44% CD8+ splenocytes), and cytotoxic (91.6±3.6% *in vivo* killing of S9I pulsed splenocytes). Antibodies to CSP were also elicited (anti-(QGPGAP)_2_ Ab titer of 2800±1567). This NILV regimen afforded some protection against malaria, with 37.5 and 62.5% sterile protection in two independent trials. The experimental differences between the two studies were (i) different strains of BALB/c mice, (ii) various batches of vectors, which were primarily characterized for their p24 content and might have slightly differed in quality and titer and finally (iii) parasites from the same strain but from different batches. The difficulty to standardize the infectivity of sporozoites stocks used for challenge is most probably the main reason for the low reproducibility in protection. Protection was long-lasting with 42.8% sterile protection afforded six months after the last immunization.

What were the differences between fully *versus* partially protected mice (no *versus* delayed and reduced parasitemia)? Analysis of post-challenge immunity revealed that the CD8+ T cell responses to CSP (but not anti-CSP Abs) correlate with protection against malaria. There is no doubt that the challenge itself boosted vaccine-induced immune responses. Post-challenge immunity is expected to reflect pre-challenge vaccine-induced responses, but it is unlikely that cells measured one month post-challenge were directly involved as effectors in the clearance of the infected hepatocytes a few days after challenge. Fully protected mice showed a stronger recalled immunity than partially protected mice one month after challenge (although it was not significant). This is consistent with a threshold of protective memory CD8+ T cells to exceed [20], [64], [65]. Whether the cytolytic activity of NILV-induced CD8+ T cells is key to protection and/or whether the secretion of IFNg and/or TNFa plays a central role remains to be determined [21], [66], [67], [68].

There were no major quantitative differences in the immune responses to CSP after NILV and RAS immunizations, but RAS immunizations were more protective than NILV and led, as expected after 3 injections, to 100% sterile protection. Differential effector mechanisms could be involved in protection after RAS and NILV immunizations. The discrepancy between immunity and protection could also be related to the induction of protective immunity to non-CSP Ags by RAS only. Although CSP-specific T cells dominate [63], the importance of non-CSP Ags in protection was highlighted in several recent studies. Some protection was reported in mice transgenic for *Py* CSP and thus tolerant to it [57], as well as in mice immunized with *Plasmodium berghei* (*Pb*) RAS and challenged with a recombinant chimeric *Pb* expressing *Pf* CSP [69], [70], [71]. In addition, in humans, both the intensity of responses and the frequency of responders among protected individuals were reported to be no higher with CSP than with other tested Ags in two complementary immunomic studies [11], [72]. Finally, the superior protection provided by late-liver stage arresting genetically attenuated parasites (GAP) compared to early-liver stage GAP or RAS also underscores the importance of the breadth of the response [73].

How did NILV perform in comparison with other CSP-based vaccine candidates? Admittedly, it has been hard to generate high-level protective efficacy with vaccines encoding a single pre-erythrocytic Ag. Heterologous prime/boost strategies are generally required. Protection levels against malaria are most of the time assessed with an early challenge with sporozoites (two weeks after the last immunization). It was reported that 40% of animals immunized against *Py* CSP were protected after a single injection of HuAd5 [74], 69% after a prime/boost with DNA/NYVAC [8], 100% after a prime/boost with HuAd5/VV, 80% after a prime/boost with YFV17D/MVA. In addition, using TRAP-ME as Ag (TRAP from *Pf* fused to a multiepitope (ME) string with multiple B cell, CD4+, and CD8+ T cell epitopes from *Pb* CSP), a prime/boost with a Chimpanzee Ad63/MVA resulted in sterile protection of 100% of the immunized animals [75]. We conclude that NILV encoding CSP elicited a good duration of protection with a vaccine efficacy yet-to improve, by providing 50% (37.5–62.5%) and 42.8% sterile protection one and six months after the last immunization, respectively.

Importantly, our lentiviral vectors are derived from HIV. Multiple restrictions of HIV replication in murine cells have been described in the literature [76], [77], [78], [79]. They include blocks in the early steps of HIV replication, suggesting that LV transduction might be impaired in murine cells as compared to human cells. Thus mice might not be the best animals to assess LV immunogenicity and predict human responsiveness to LV vaccines. The use of similar doses of LV in mice and monkeys [30] is an indirect piece of evidence for a reduced LV transduction efficiency in murine cells.

Our goal was to provide a comprehensive assessment of the protective efficacy of NILV against malaria. Our data are promising. They prompt us to design a novel generation of NILV, improved for their immunogenicity [80], as well as to identify new protective Ags to be added to CSP in a multigenic vaccine [81]. With the upcoming improvements, up-scaling of lentiviral vector production and their stable conservation at 4°C after lyophilisation, we believe that this novel vaccine strategy could impact public health in the malaria domain.

## Methods

### Animals

Six-week-old female Balb/c were purchased from Harlan Laboratories (Gannat, France). Because of a shortage from the breeder company, two strains of BALB/c mice with the same origin were used. BALB/cOlaHsd were used for the immunogenicity studies and for the protective efficacy study comparing NILV and RAS (**Figures 2** **,** **3** **,** **4** **,** **5** **,** **6**), while BALB/cAnNHsd were used to confirm the protective efficacy induced by NILV one month after the last immunization (**Figure 7**) and assess the duration of protection (**Figure 8**).

### Ethics Statement

All animal experiments were conducted in accordance with guidelines established by the French and European regulations for the care and use of laboratory animals (Décrets 87–848, 2001–464, 2001–486 and 2011–131 and European Directive 2010/63/UE). The Institut Pasteur is in compliance with Standards for Human Care and Use of Laboratory Animals and is accredited by the US National Institut of Health Office of Laboratory Animal Welfare (OLAW) (Animal Welfare Assurance Number: A5476-01). Every effort was made to minimize suffering, as described in the Guide for the ethical evaluation of experiments using laboratory animals edited by the GIRCOR (Groupe Interprofessionnel de Réflexion et de Communication sur la Recherche). This study was approved by the Regional Committee on Ethics and Animal Experimentation (CREEA) Ile de France Paris 1 (protocol #2011-0007) and ASB holds the authorization for animal experimentation #A-75-1747.

### Vector Plasmid Construction

The vector plasmid carrying a synthetic *Homo sapiens* codon optimized form of Py CSP (Geneart) (pTRIP.ieCMV. Py CSP co.WPRE), with a Kozak consensus sequence and ATG start codon at 5′ flanking site and TGA stop codon at 3′ flanking site, was generated by replacing the eGFP sequence from pTRIP.ieCMV.eGFP.WPRE after Bgl2/XhoI digestion with the Py CSP co sequence. The comparison between the wild-type and codon-optimized sequences is shown in **Figure S1**.

### Envelope Expression Plasmids Construction

Mammalian codon-optimized synthetic genes (GeneArt) encoding glycoproteins from the following Vesiculovirus were cloned into a pVAX1 plasmid (Invitrogen): Vesicular Stomatitis Virus Indiana serotype (GenBank FW591952), New Jersey serotype (GenBank FW591956) and Cocal virus (GenBank: AF045556.1).

### Lentiviral Vector Particles Production

HIV-1 derived vector particles were produced by transient calcium phosphate co-transfection of HEK 293 T cells (ATCC) with the vector plasmid pTRIP, an envelope expression plasmid (encoding the glycoprotein from VSV, serotype Indiana (IND) or New Jersey (NJ), or the glycoprotein from Cocal virus) and the p8.7 or pD64V encapsidation plasmid for the production of ILV or NILV particles respectively (as shown in **Figure 1**). The p24 (encoded by HIV-1 Gag from the encapsidation plasmid) content was quantified by ELISA and expressed as ng p24/µL (physical characterization). Vector gene transfer capacity was determined by quantitative PCR after transduction of P4-CCR5 cells (which are CD4+ CXCR4+ and CCR5+ HeLa cells carrying the LacZ gene under the control of the HIV-1 long terminal repeat (LTR) promoter [82]) in the presence of aphidicolin (Sigma) as previously described [47] and was expressed as transduction unit (TU)/µL of vector (functional characterization).

### Parasites

Immunization and infection were performed with the non-lethal strain *Plasmodium yoelii* (Py) 17XNL, which was maintained by alternate cyclic passages in *Anopheles stephensi* and Balb/c mice. Parasitized red blood cells were maintained as frozen stabilate. Mosquitoes were reared at the Center for Production and Infection of Anopheles (CEPIA) of the Institut Pasteur using standard procedures. Sporozoites were prepared by the Ozaki method [83]. Radiation-attenuated sporozoites (RAS) were prepared as described previously [84]. They were irradiated at the dose of 18,000 Rad on ice using a gamma irradiator (IBL637 irradiator).

### Peptides

Synthetic peptides (PolyPeptide Laboratories France) were used as Ag. S9I (Py CSP:280–288, SYVPSAEQI) and I10L (Py CSP:58–67, IYNRNIVNRL) contain a CD8+ T cell epitope and S16I (Py CSP:280–296, SYVPSAEQILEFVKQI) contains both a CD4+ and the S9I CD8+ T cell epitope [60], [61] (**Figure S1**). (QGPGAP)_2_ corresponds to the major central repeat of Py CSP which is targeted by neutralizing antibodies [85].

### NILV and RAS Mice Immunizations and Challenge

BALB/c mice were immunized by intra-peritoneal (IP) or intramuscular (IM) injection of NILV. Doses were expressed as ng p24/mouse and/or TU/mouse. BALB/c mice were immunized by intravenous (IV) injection of 50,000 RAS (in the retro-orbital vein). RAS were injected immediately after irradiation. Several groups of mice were immunized in parallel so as to follow the specific B and T cells responses in blood over time in one group, to study responses in spleen and liver in another group at necropsy and to analyze the cytotoxic activity *in vivo* in a third group. Animals included in the protective efficacy studies differed from those included in the immunogenicity studies. They were immunized and challenged but not used for pre-challenge immune responses characterizations.

Challenge experiments consisted in the IV injection of 500 Py 17XNL sporozoites in the retro-orbital vein. Thin blood smears were stained with Giemsa and screened for the presence of parasites in red blood cells.

### Single Cell Suspensions Preparation

After euthanasia with CO_2_, the liver was perfused *in situ* through the portal vein with PBS to remove circulating blood. Liver was then dissected out and transferred into HBSS complemented with 5% FCS and gently squished on a 100-µm cell strainer. Parenchymal cells (pellet) were removed by centrifugation at 50 g for 5 min. After a single wash by centrifugation at 300 g, T cells were further enriched using a 35% Percoll (Sigma) RPMI solution and centrifugation at 1360 g for 25 minutes [86]. Red blood cells from spleen were lysed using IOTest 3 lysing solution **(**Beckman Coulter).

### Tetramer Staining

Whole blood for longitudinal follow-up and splenocytes or liver cells at necropsy were stained with an anti-mouse CD8a mAb conjugated to APC (clone 53-6.7, BD Biosciences) and the S9I-K^d^ tetramer-PE (Class I iTAG™ MHC custom tetramer, Beckman Coulter, Fullerton, USA).

### Elispot Assay

Nitrocellulose microplates (MAHA S4510, Millipore) were coated with capture antibody (Mouse IFNg Elispot pair, BD Pharmingen) and blocked with complete medium. Cells were cultured at the concentration of 0.2 million/well. They were incubated with 2 µg/ml of S9I, I10L or S16I peptides. Eigtheen hours later, spots were revealed with the biotine-conjugated antibody (Mouse IFNg Elispot pair, BD Pharmingen) followed by streptavidin-AP (Roche) and BCIP/NBT substrate solution (Promega). Spots were counted using a Bioreader 2000 (Biosys, Karben, Germany). Mean number of IFNg spots-forming-cells (SCF) per million cells was calculated from triplicate wells after substracting the one from control wells (cultured in medium without peptide).

### Intracellular Cytokines Staining (ICS)

Splenocytes (2 millions/well) were cultured in the presence of the S9I peptide (2µg/ml final) and anti-CD28 NA/LE MAb (1µg/ml final, clone 37.51, BD Biosciences) for 1 hour. Then Brefeldin A from *Penicillium brefeldianum* (2µg/ml final, Sigma) was added for 5 hours culture and cells were surface-stained for CD8a expression (anti-CD8a-PerCP, clone 53-6.7, BD Pharmingen) and intracellular-stained for IFNg, (anti-IFNg-FITC, clone XMG1.2, BD Pharmingen), IL2 (anti-IL2-PE clone JES6-5H4, eBiosciences) and TNFa (anti-TNFa-APC**,** clone MP6-XT22, eBiosciences). Flow-cytometry acquisition and analysis were done with a CyAn2 ADP analyser (Beckman Coulter, UK) equipped with Summit2 and with FlowJo respectively.

### *In vivo* Cytotoxic Assay

For target cells preparation, splenocytes from naive mice were labeled with two concentrations (5 and 1 µM) of CFSE (carbosyfluorescein-diacetate succinimydel ester, Vybrant CFDA-SE cell-tracer kit, Molecular Probes). Splenocytes labeled with the high concentration of CFSE were also pulsed with 5 µg/ml of the S9I peptide. Each mouse received a mix of 10E+07 CFSE-labeled cells containing an equal number of S9I pulsed and unpulsed cells through the retro-orbital vein. After 15 h, single-cell suspensions from spleen were analyzed by flow cytometry. The disappearance of S9I-pulsed cells was determined by comparing the ratio of pulsed to unpulsed populations in immunized versus naive mice. The percentage of specific killing was established according to the following calculation: (1-((CFSE^low^ naive/CFSE^high^ naive)/(CFSE^low^ immunized/CFSE^high^ immunized)))*100.

### Anti-CSP Antibody Response

NUNC Maxisorps plates were coated with the (QGPGAP)_2_ peptide diluted in PBS as described previously. After incubation with serial dilutions of serum from immunized animals, the presence of specific Abs was revealed using a Peroxidase-Conjugated AffiniPure Goat anti-mouse IgG (Jackson Immuno Research Laboratory) and OPD substrate (Sigma). OD was measured at 492 nm with a Victor (Perkin-Elmer). Specific antibody titers were defined as the reciprocal serum dilution giving an optical density equal to 2 times the background obtained with a pool of serum samples from naive mice (2OD = 0.126, 0.164 and 0.162 for Figures 2H, 4E and 6E respectively).

### Statistics

Non-parametric tests were used (Prism, GraphPad). To compare two groups, the Mann-Whitney test was used. To compare more than two groups, the Kruskal-Wallis test was used, followed by a Dunn’s post-test to compare all pairs. The Spearman test was used to study correlations between immunity and parasitemia, and linear regression was used to find out whether immune responses predict the % of parasitized red blood cells. Only statistically significant p values are indicated.

## Supporting Information

Figure S1 **Sequence of the codon-optimized CSP synthetic gene.** The CSP synthetic *Homo sapiens* codon-optimized DNA sequence (GeneArt) is shown in blue and compared with the wild-type DNA sequence in red (GenBank: J02695.1), which is T and A rich. There is 51% similarity between both DNA sequences. The amino acid sequence is also shown in green (UniProtKB/Swiss-Prot: P06914.1). The peptides containing a CD8+ T cells epitope used in the study, *Py* CSP S9I and I10L, as well as the major central repeat were underlined.
(DOC)

Figure S2 **The lower immunogenicity of NILV as compared to ILV is also true for an unrelated Ag, SIV GAG.** C57BL/6 mice (n = 3/group) were immunized IP with 900 ng p24 of NILV or ILV carrying a wild-type form of the gene encoding SIVmac239 GAG. T cell responses were evaluated eleven days later by IFNg elispot after restimulation of splenocytes with the AL11 peptide, which contains the CD8^+^ T cell immunodominant epitope. Means + SD are shown.
(TIF)
