# Supplementary material for: A Nonintegrative Lentiviral Vector-Based Vaccine Provides Long-Term Sterile Protection against Malaria
Source: PLoS One. 2012 Nov 2;7(11):e48644. doi: 10.1371/journal.pone.0048644 (PMC3487763; doi:10.1371/journal.pone.0048644)
Supplement: Figure S1 — Sequence of the codon-optimized CSP synthetic gene. The CSP synthetic Homo sapiens codon-optimized DNA sequence (GeneArt) is shown in blue and compared with the wild-type DNA sequence in red (GenBank: J02695.1), which is T and A rich. There is 51% similarity between both DNA sequences. The amino acid sequence is also shown in green (UniProtKB/Swiss-Prot: P06914.1). The peptides containing a CD8+ T cells epitope used in the study, Py CSP S9I and I10L, as well as the major central repeat were underlined. (DOC) [file pone.0048644.s001.doc]

**ATGAAGAAATGCACCATCCTGGTGGTGGCCAGCCTGCTGCTGGTCGATAGCCTGCTGCCCGGCTACGGCCAGAATAAGAGC**

**ATGAAGAAGTGTACCATTTTAGTTGTAGCGTCACTTTTATTAGTTGATTCTCTACTTCCAGGATATGGACAAAATAAAAGT**

**M K K C T I L V V A S L L L V D S L L P G Y G Q N K S**

**GTGCAGGCCCAGCGGAACCTGAACGAGCTGTGCTACAACGAGGAAAACGACAACAAGCTGTACCACGTGCTGAACAGCAAG**

**GTCCAAGCCCAAAGAAACTTAAACGAGCTATGTTACAATGAAGAAAATGATAATAAATTGTATCACGTCCTTAACTCGAAG**

**V Q A Q R N L N E L C Y N E E N D N K L Y H V L N S K**

**AACGGCAAGATCTACAACCGGAACATCGTGAACAGGCTGCTGGGCGACGCTCTGAACGGCAAGCCCGAGGAAAAGAAGGAC**

**AATGGAAAAATATACAATCGAAATATAGTCAACAGATTACTTGGCGATGCTCTCAACGGAAAACCAGAAGAAAAAAAAGAT**

**N G K I Y N R N I V N R L L G D A L N G K P E E K K D**

**GACCCCCCCAAGGACGGCAACAAGGACGACCTGCCCAAAGAAGAGAAGAAAGACGATCTGCCTAAAGAGGAAAAAAAAGAC**

**GATCCCCCAAAAGATGGCAACAAAGATGATCTTCCAAAAGAAGAAAAAAAAGATGATCTTCCAAAAGAAGAAAAAAAAGAT**

**D P P K D G N K D D L P K E E K K D D L P K E E K K D**

**GATCCTCCTAAGGACCCCAAGAAGGATGACCCTCCTAAAGAGGCCCAGAACAAGCTGAACCAGCCCGTGGTGGCCGACGAG**

**GATCCCCCAAAAGATCCTAAAAAAGATGATCCACCAAAAGAGGCTCAAAATAAATTGAATCAACCAGTAGTGGCAGATGAA**

**D P P K D P K K D D P P K E A Q N K L N Q P V V A D E**

**AACGTGGATCAGGGACCTGGCGCCCCTCAGGGCCCAGGCGCTCCACAGGGACCCGGGGCACCCCAGGGGCCTGGGGCCCCA**

**AATGTAGATCAAGGGCCAGGAGCACCACAAGGGCCAGGAGCACCACAAGGACCAGGAGCACCACAGGGTCCAGGAGCACCA**

**N V D Q G P G A P Q G P G A P Q G P G A P Q G P G A P**

**CAGGGACCAGGGGCTCCTCAGGGCCCTGGCGCACCTCAGGGGCCAGGGGCCCCTCAGGGGCCTGGCGCTCCCCAGGGACCT**

**CAAGGACCAGGAGCACCACAAGGACCAGGAGCACCACAAGGTCCAGGAGCACCACAGGGTCCAGGAGCACCACAGGGTCCA**

**Q G P G A P Q G P G A P Q G P G A P Q G P G A P Q G P**

**GGCGCACCACAGGGCCCTGGGGCTCCCCAGGGCCCAGGCGCCCCTCAGGGACCAGGCGCACCCCAGGGACCCGGCGCTCCT**

**GGAGCACCACAAGGACCAGGAGCACCACAGGGGCCAGGAGCACCACAAGGACCAGGAGCACCACAAGGACCAGGAGCACCA**

**G A P Q G P G A P Q G P G A P Q G P G A P Q G P G A P**

**CAGGGACCTGGGGCTCCACAGGGGCCAGGCGCACCACAGGAACCTCCCCAGCAGCCTCCTCAGCAGCCACCCCAGCAGCCC**

**CAGGGGCCAGGAGCACCACAAGGGCCAGGAGCACCACAAGAACCACCCCAACAACCACCCCAACAACCACCACAACAGCCA**

**Q G P G A P Q G P G A P Q E P P Q Q P P Q Q P P Q Q P**

**CCTCAGCAGCCTCCTCAGCAGCCCCCACAGCAGCCTCCACAGCAGCCTAGACCCCAGCCCGACGGCAATAACAACAACAAT**

**CCACAACAGCCACCACAACAGCCACCACAACAGCCACCACAACAACCACGCCCACAGCCAGATGGTAATAACAACAATAAC**

**P Q Q P P Q Q P P Q Q P P Q Q P R P Q P D G N N N N N**

**AATAACAACGGCAACAACAACGAGGACAGCTACGTGCCCAGCGCCGAGCAGATCCTGGAATTCGTGAAGCAGATCAGCAGC**

**AATAATAATGGTAATAATAATGAAGATTCTTATGTCCCAAGCGCGGAACAAATACTAGAATTTGTTAAACAGATAAGTAGT**

**N N N G N N N E D S Y V P S A E Q I L E F V K Q I S S**

**CAGCTGACCGAAGAGTGGAGCCAGTGCAGCGTGACATGCGGCTCTGGCGTGAGAGTGCGGAAGCGGAAGAACGTGAACAAG**

**CAACTCACAGAGGAATGGTCTCAATGTAGTGTAACCTGTGGTTCTGGTGTAAGAGTTAGAAAACGAAAAAATGTAAACAAG**

**Q L T E E W S Q C S V T C G S G V R V R K R K N V N K**

**CAGCCCGAGAACCTGACCCTGGAAGATATCGACACCGAGATCTGCAAGATGGACAAGTGCAGCAGCATCTTCAACATCGTG**

**CAACCAGAAAATTTGACCTTAGAGGATATTGATACTGAAATTTGTAAAATGGATAAATGTTCAAGTATATTTAATATTGTA**

**Q P E N L T L E D I D T E I C K M D K C S S I F N I V**

**TCCAACAGCCTGGGCTTCGTGATCCTGCTGGTGCTGGTGTTCTTCAACTGA**

**AGCAATTCATTAGGATTTGTAATATTATTAGTATTAGTATTCTTTAATTAA**

**S N S L G F V I L L V L V F F N ***

**Figure S1**
