# Supplementary figures and images for: A Nonintegrative Lentiviral Vector-Based Vaccine Provides Long-Term Sterile Protection against Malaria
Source: PLoS One. 2012 Nov 2;7(11):e48644. doi: 10.1371/journal.pone.0048644 (PMC3487763; doi:10.1371/journal.pone.0048644)

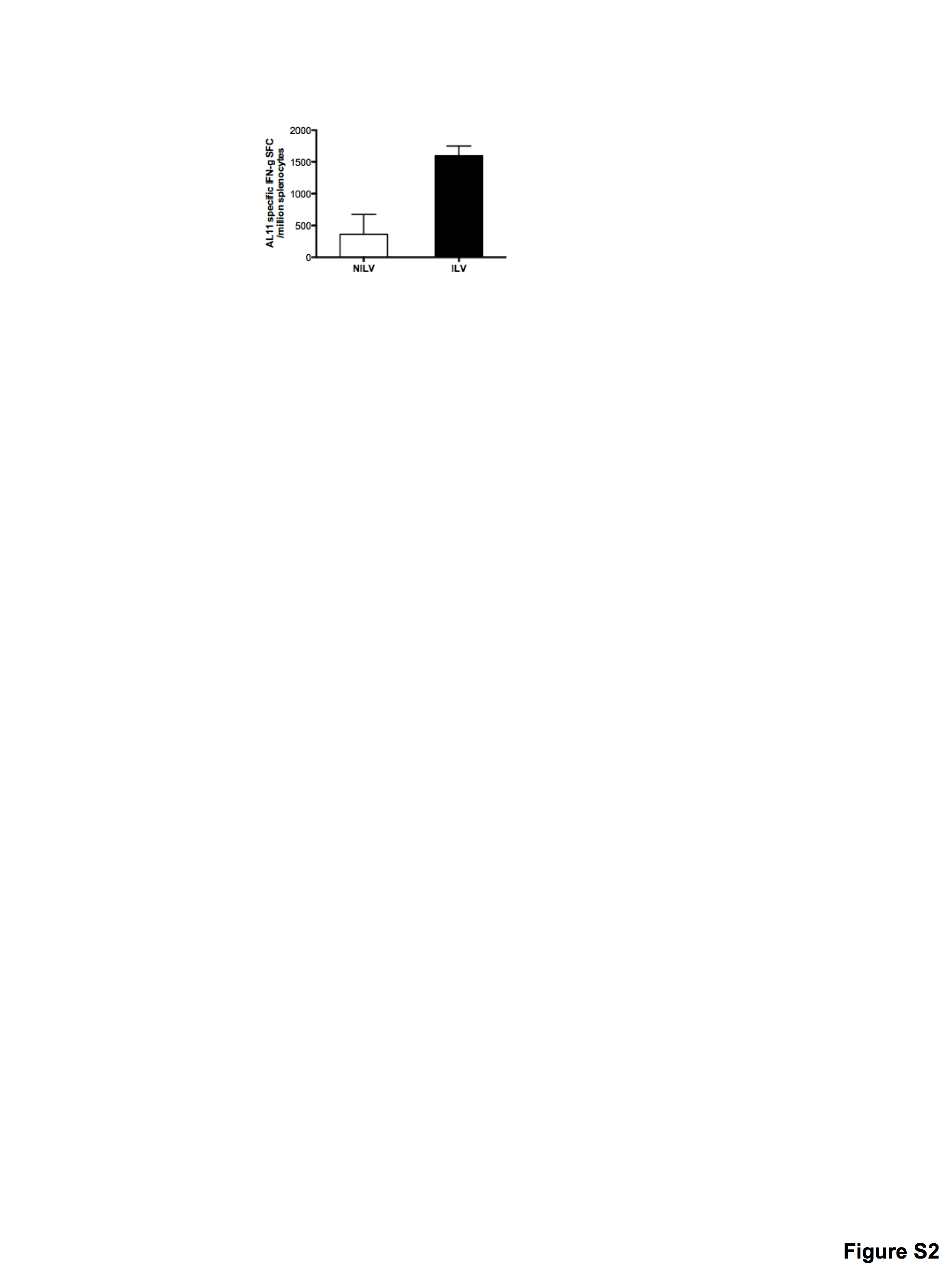

Supplement: Figure S2 — The lower immunogenicity of NILV as compared to ILV is also true for an unrelated Ag, SIV GAG. C57BL/6 mice (n = 3/group) were immunized IP with 900 ng p24 of NILV or ILV carrying a wild-type form of the gene encoding SIVmac239 GAG. T cell responses were evaluated eleven days later by IFNg elispot after restimulation of splenocytes with the AL11 peptide, which contains the CD8+ T cell immunodominant epitope. Means + SD are shown. (TIF) [file pone.0048644.s002.tif]
